# Estimability study on the age of toddlers’ gait development based on gait parameters

**DOI:** 10.1038/s41598-023-30039-7

**Published:** 2023-02-20

**Authors:** Chisa Tsuyuki, Haruna Hiraga, Motoki Sudo, Tomoya Ueda, Kanako Seo, Masayuki Minatozaki, Yuko Fukuda, Yasuyuki Okuda, Hiroyuki Iwasaki, Hisashi Naito, Dajiang Lu

**Affiliations:** 1 Tokyo Research Laboratories, Kao Corporation, 2-1-3 Bunka, Sumida-Ku, Tokyo, 131-8501 Japan; 2 Graduate School of Health and Sports Science, Juntendo University, 1-1 Hirakagakuendai, Inzai, Chiba 270-1695 Japan; 3 Tochigi Research Laboratories, Kao Corporation, 2606 Akabane, Ichikai-machi, Haga-gun, Tochigi, 321-3497 Japan; 4 Jujo Pediatric Clinic, 3-22-8-101 Kamijujo, Kita-ku, Tokyo, 114-0034 Japan; 5 Department of Human Sports Science, Shanghai University of Sport, Shanghai, 200438 China

**Keywords:** Biomechanics, Human behaviour

## Abstract

In the first few years of toddlers’ locomotion, various gait parameters improve gradually and dynamically with gait development. Therefore, in this study, we hypothesized that the age of gait development, or the level of gait development with age as its indicator, can be estimated from several gait parameters related to gait development, and investigated its estimability. In total, 97 healthy toddlers aged about 1–3 years participated in the study. All five selected gait parameters showed a moderate or higher correlation with age, but the duration with a large change and the strength of the association with gait development varied for each gait parameter. Multiple regression analysis was performed using age as the objective variable and five selected gait parameters as explanatory variables, and an estimation model (*R*^2^ = 0.683, adjusted *R*^2^ = 0.665) was created. The estimation model was verified using a test dataset separate from the training dataset (*R*^2^ = 0.82, *p* < 0.001). It was suggested that the age of gait development could be estimated from gait alone. Gait analysis based on empirical observations may reduce the need for skilled observers and their potential variability.

## Introduction

Walking is a basic movement in daily life and a useful functional vital sign^1^ that can assess an individual’s current health condition and risk of future illness. The same is true for toddlers who have just started walking. There are cases where gross motor control, such as walking, is used to estimate the prognosis of cerebral palsy in young children^2^.

Independent locomotion for toddlers is a very important ability in motor development. It is a valuable evaluation indicator that can assess growth and development through external observation. Therefore, many researchers have revealed a relationship between toddlers’ gait and development through external observations. For example, Okamoto et al.^3^ reported that gait maturity begins at approximately 12 months of age and develops gradually during the first 3 years of life through a series of phases, namely newborn stepping, toddler-supported walking, and toddler-independent walking, toward mature walking. In the first few months after the onset of independent walking, very large changes occur in gait parameters, especially spatiotemporal parameters, joint kinematics, and dynamics^4,5^.

Therefore, to elucidate this remarkable mechanism of gait development in toddlers, various studies have been conducted since the 1940s. Recently, Ledebt^6^ and Okamoto et al.^3^, using electromyography, force plates, and video cameras, revealed that toddlers in the early stages of gait acquisition adopt a high-guard posture in which the upper limbs are raised above the shoulder when walking. Subsequently, over the course of several months, they gradually adopt the position of a middle- and low-guard posture by lowering their arms, and the alternating arm-swing seen in adult gait appears. Sutherland et al.^7^, using high-velocity images, Graf-Pen sonic digitizers, and plotters, showed that toddlers in the early stages of gait acquisition walked while maintaining stability by enlarging the base of support with a broad step width, and mature gait is established around 3 years old. Thus, the gait development process of toddlers until they acquire a mature gait has been gradually clarified from various perspectives using different analysis methods.

Based on these, we hypothesized that by combining several gait parameters that have been reported to be associated with gait development in such previous studies, we can estimate the age of gait development, or the level of gait development with age as its indicator, only from their gait, just as the age of blood vessels can be estimated from the suppleness and hardness of blood vessels. If the level of gait development of a toddler can be evaluated using objective and quantitative indicators such as the age of gait development, it will be possible to grasp the gait development status of toddlers relatively easily even for amateurs who are not experts. However, it is challenging to collect walking data on toddlers who do not walk as instructed, and there are fewer examples of gait studies in young children than in adults. Therefore, accurate gait data of toddlers collected by a unified model that comprehensively captures independent walking in toddlers^4,8^ is limited. Furthermore, there is currently neither an adequate standard database to provide the necessary reference values for assessing the normal developmental stage of gait in toddlers nor previous studies that clarify the strength of the association between gait parameters and toddlers’ gait development.

Therefore, this study aimed to estimate the age of gait development from toddlers’ gait and conducted a cross-sectional survey of 97 toddlers aged about 1–3 years old, which is said to be the mature period of gait^7,9,10^. In the first half of the study, we confirmed that the developmental tendencies with respect to age in five gait parameters (hand height^3,6^, knee-to-knee distance^7^, waist height^8^, trunk sway, and walk ratio^11^), which have been reported to be associated with gait development in previous studies, were reproduced by a unified model and the same subject. In the second half, we investigated the strength of the association between these gait parameters and toddlers’ gait development, and verified the accuracy of the estimation model of gait development age created using the strongly associated gait parameters.

## Results

### Correlation between each gait parameter and age

**Figure 1:**
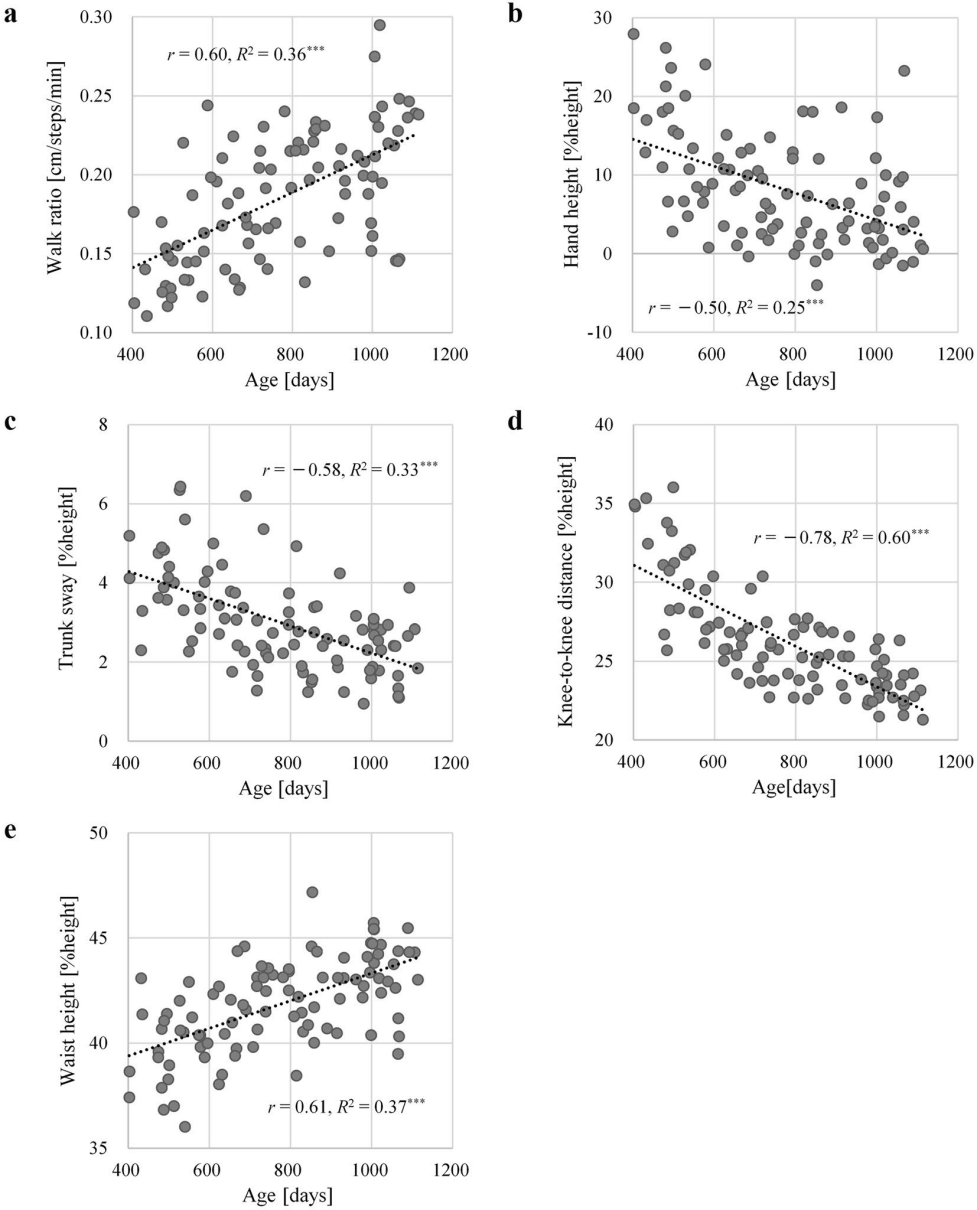
Relationship between age and each gait parameter: (**a**) walk ratio, (**b**) hand height, (**c**) trunk sway, (**d**) knee-to-knee distance, and (**e**) waist height. [%height] indicates the height ratio. *r*: correlation coefficient, *R*^2^: coefficient of determination, *p*: p-value, ***p < 0.001. Dashed line represents the trend line for the slope.

Figure 1 shows the correlation coefficient between the scatterplot and Pearson’s correlation coefficient of each gait parameter with respect to age. The correlation coefficient was either moderate or greater for all gait parameters and was significant (*p* < 0.001). Age and knee-to-knee distance were the most strongly correlated (*r* = − 0.78, *R*^2^ = 0.60), followed by age and waist height (*r* = 0.61, *R*^2^ = 0.37), age and walk ratio (*r* = 0.60, *R*^2^ = 0.36), age and trunk sway (*r* = − 0.58, *R*^2^ = 0.33), and age and hand height (*r* = − 0.50, *R*^2^ = 0.25). Among the five gait parameters, walk ratio and waist height showed a positive correlation with age, and the hand height, trunk sway, and knee-to-knee distance showed a negative correlation.

Table 1 shows the results of comparing the measurement results of each gait parameter by age into three groups (A: 13–21 months, B: 22–29 months, C: 30–37 months). Group A showed significant differences in all gait parameters compared to the Groups B and C. The only gait parameter that was significantly different between Groups B and C was knee-to-knee distance.

**Table 1:** Measurement results of gait parameters for each age group.

N = 97 (49 boys, 48 girls)	Group	Mean	SD	95% CI		RM ANOVA		Bonferroni’s post-hoc test
				Lower	Upper	*p*	*η*^2^	
Walk ratio (cm/steps/min)	A	0.16	0.03	0.15	0.17	***	0.286	A < B*** A < C***
	B	0.19	0.03	0.18	0.20			
	C	0.21	0.04	0.20	0.22			
Hand height (%height)	A	12.66	7.11	10.18	15.14	***	0.224	A > B*** A > C***
	B	6.11	5.75	4.00	8.22			
	C	5.28	5.93	3.14	7.41			
Trunk sway (%height)	A	3.93	1.09	3.55	4.31	***	0.329	A > B*** A > C***
	B	2.72	1.14	2.30	3.13			
	C	2.30	0.78	2.01	2.58			
Waist height (%height)	A	39.97	1.80	39.34	40.60	***	0.411	A < B*** A < C***
	B	42.43	1.76	41.79	43.08			
	C	43.27	1.61	42.69	43.86			
Knee-to-knee distance (%height)	A	29.34	3.38	28.17	30.52	***	0.499	A > B*** A > C*** B > C***
	B	25.63	1.96	24.91	26.35			
	C	23.65	1.44	23.13	24.17			

Group A, 13–21 months (N = 34); Group B, 22–29 months (N = 31); Group C, 30–37 months (N = 32). [%height] indicates the height ratio.

Data are presented as means.

SD: standard deviation, CI: confidence interval, *p*: p-value, *η*^2^: effect size and Bonferroni’s post-hoc test.

***p < 0.001.

### Multiple regression analysis of five gait parameters and age

Table 2 summarizes the results of multiple regression analysis using a training dataset of 78 toddlers (80%) with the objective variable as age and the explanatory variable as the above five gait parameters. Models I, II, and III refer to the forced entry, backward elimination, and forward inclusion methods, respectively. Model II, which had the highest coefficient of determination, improved the accuracy of multiple regression by employing four gait parameters other than hand height (*R*^2^ = 0.683, adjusted *R*^2^ = 0.665). From the value of the standardized partial regression coefficient β, it was observed that the gait parameter that contributed the most to accuracy improvement of the multiple regression equation was knee-to-knee distance, followed by waist height, walk ratio, and trunk sway. The Model II multiple regression equation (Eq. X) with the highest coefficient of determination is shown as follows:

X $${\text{Age in days }} = \, (0.{86} \times {1}0^{{3}} ) \, + \, (0.{78} \times {1}0^{{3}} ) \times {\text{Walk ratio}} - {23}.{22} \times {\text{Trunk sway }} + { 15}.{97} \times {\text{Waist height}} - {31}.{6}0 \times {\text{Knee-to-knee Distance}}{.}$$

**Table 2:** Results of the multiple regression analysis with age as the dependent variable.

Model		B	β	95% CI (Lower)	95% CI (Upper)	*p*
I	Constant	0.81 × 10^3^	–	0.09 × 10^3^	1.54 × 10^3^	*
	Walk ratio	0.81 × 10^3^	0.17	0.04 × 10^3^	1.59 × 10^3^	*
	Hand height	1.18	0.04	− 3.44	5.79	0.612
	Trunk sway	− 22.70	− 0.15	-48.55	3.15	0.084
	Waist height	17.10	0.20	2.00	32.21	*
	Knee-to-knee distance	− 32.25	− 0.55	− 42.90	− 21.60	***
II	Constant	0.86 × 10^3^	–	0.16 × 10^3^	1.56 × 10^3^	*
	Walk ratio	0.78 × 10^3^	0.16	0.02 × 10^3^	1.54 × 10^3^	*
	Hand height	–	–	–	–	–
	Trunk sway	− 23.22	− 0.15	-48.85	2.41	0.075
	Waist height	15.97	0.19	1.60	30.34	*
	Knee-to-knee distance	− 31.60	− 0.54	− 41.88	− 21.31	***
III	Constant	0.78 × 10^3^	–	67.50	1.50 × 10^3^	*
	Walk ratio	–	–	–	–	–
	Hand height	–	–	–	–	–
	Trunk sway	–	–	–	–	–
	Waist height	23.47	0.27	10.04	36.91	***
	Knee-to-knee distance	− 37.60	− 0.64	− 46.79	− 28.41	***

Model I: forced entry method. Model II: backward elimination method. Model III: forward inclusion method.

B: partial regression coefficient, β: standardized partial regression coefficient, CI: confidence interval, *p*: p-value.

*p < 0.05; ***p < 0.001.

**Figure 2:**
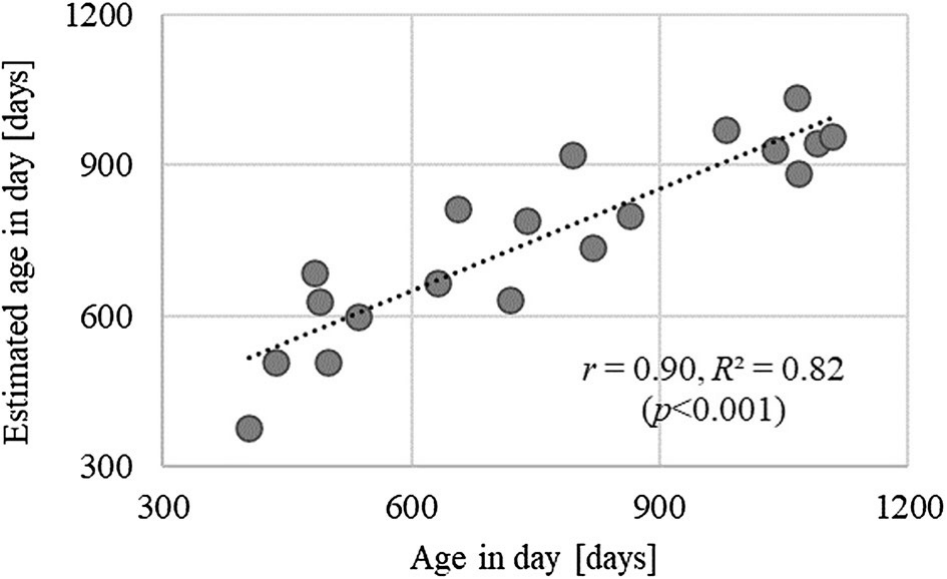
Relationship between actual age and age of gait development calculated using an estimation model. *r*: correlation coefficient, *R*^2^: coefficient of determination, *p*: p-value. Dashed line represents the trend line for the slope.

Subsequently, the accuracy of Model II was verified using the test dataset. Figure 2 shows the relationship between the actual ages of the 19 toddlers in the test dataset and the ages estimated from the gait parameter using Eq. (X). The estimates showed a significant positive correlation with actual age (*r* = 0.90, *R*^2^ = 0.82, *p* < 0.001).

## Discussion

The purpose of this study was to estimate the age of gait development from toddlers’ gait. We investigated the strength of the association between toddlers’ gait development and five gait parameters that have been reported to be associated with gait development in previous studies. Then, the accuracy of an estimation model of gait development age created using gait parameters that were strongly associated was verified. From the results of the correlation analysis, the five gait parameters showed a moderate or greater correlation with age. The gait parameter with the strongest correlation with age was the knee-to-knee distance (*r* = − 0.78, *R*^2^ = 0.60, *p* < 0.001), and the one with the weakest correlation was hand height (*r* = − 0.50, *R*^2^ = 0.25, *p* < 0.001). In addition, as a result of comparison between three age groups, significant differences were confirmed in all gait parameters between the youngest group and the other two groups (middle and oldest). On the other hand, the only gait parameter with a significant difference between the middle and oldest groups was knee-to-knee distance. Multiple regression analysis using training data demonstrated that the gait parameter that contributed most to improving the accuracy of the multiple regression equation was knee-to-knee distance, followed by waist height, walk ratio, and trunk sway. Hand height contributed less to accuracy improvement of the multiple regression equation than the other four gait parameters. Using a test dataset, age was estimated from four gait parameters excluding hand height based on the results of multiple regression analysis, and the estimates showed a significant positive correlation with actual age (*r* = 0.90, *R*^2^ = 0.82, *p* < 0.001).

In this study, knee-to-knee distance showed a significant negative correlation with age and was the gait parameter that contributed the most to improving the accuracy of the multiple regression equation. Regarding knee-to-knee distance or leg opening, which was significantly associated with age in this study, Sutherland et al.^7^ showed that toddlers in the early stages of gait acquisition walked while maintaining stability by increasing step width, thereby enlarging their support base. Furthermore, they stated that maturity was manifested by increased limb length and stability and was well established in toddlers approximately 3 years old. Additionally, in this study, knee-to-knee distance was the only indicator showing significant differences among all three age groups. As described above, knee-to-knee distance is unlikely to cause accidental changes unless balance ability is developed; in other words, it is a stable index. In addition, significant improvement can be seen not only in the first few months of gait onset but also from 1 to 3 years of age. Therefore, it is thought that knee-to-knee distance greatly contributed to improving the accuracy of the multiple regression equation.

Next to knee-to-knee distance, the indicators that contributed to improving the accuracy of the multiple regression equation were waist height and walk ratio, both of which were significantly positively correlated with age. Hallemans et al*.*^8^ stated that toddlers are unable to extend the knee joint when walking due to insufficient muscle development and that the knee joint flexion angle is larger in both the stance and swing phases compared to that of adults; however, the flexion positions of the hip, knee, and ankle decreased significantly at approximately 5 months after the start of walking with the development of balance ability. Yaguramaki et al*.*^12^ also showed that movements around the knees and ankles changed significantly months after the start of walking. In other words, the waist height increases with respect to height by allowing flexion and extension of the knees and ankles as the child grows. According to Cheron et al*.*^11^, the proficiency in walking, especially cadence and step length, along with upright posture, trunk stability, and dynamic control of the limb area, improved in the first few months of independent walking and then developed gradually over 7–8 years or more. In this study as well, waist height and walk ratio were significantly different among the three age groups. However, no significant difference was shown in between the middle and oldest groups. In other words, waist height and walk ratio were indicators that developed significantly during the first few months to about 1 year in gait onset compared to the knee-to-knee distance that developed significantly from about 1–3 years old. Therefore, we considered that waist height and walk ratio contributed less than knee-to-knee distance.

Trunk sway showed a significant negative correlation with age and contributed to improving the accuracy of the multiple regression equation, next to knee-to-knee distance, waist height, and walk ratio. According to Cheron et al.^11^, head and trunk sway improved significantly in the first few months of independent walking. On the other hand, Assaiante et al.^13^ confirmed that hip stabilization occurred at approximately the first week of independent walking and preceded shoulder and head stabilization, and that hip movement was followed by shoulder and head movement. Iosa et al.^14^ also reported that gait stability can be achieved with the stabilization of the head from approximately 7 years of age. In this study too, through a comparison of three age groups, we confirmed that trunk sway developed significantly, especially in the early stages of gait onset. These results suggest that trunk sway contributes to improving the accuracy of the multiple regression equation in this study of 1- to 3-year-old toddlers. However, although the contribution of trunk sway was close to waist height and walk ratio, no significance was confirmed. This is presumably to be partly due to the fact that trunk sway, which expresses movement of the torso, is not an index that changes as dynamically as the waist height and walk ratio, which are defined based on the movement of the limbs. Although there are still issues to be examined in the future, trunk sway is an indicator that is relatively easy to observe visually and shows a significant correlation with age; therefore, it may be a more useful indicator depending on the selected parameters.

On the other hand, among the five gait parameters, hand height showed a moderate correlation with age (*r* = − 0.50). However, it did not improve the accuracy of the multiple regression equations. In previous studies, toddlers in the early stages of gait acquisition adopted a high-guard posture in which the upper limbs were raised above the shoulder when walking, followed by a gradual decrease in the position of the upper limbs (middle- and low-guard postures), and the reciprocal arm-swing that counterbalances trunk rotation, as observed in adults, appeared^3,6^. Thus, it can be said that hand height is a useful gait parameter to evaluate gait development. However, it is considered an index that pales compared to the five gait parameters selected in this study. The first reason for this is the possibility that the change in individual hand height has been buried in group variations. Previous studies have shown that some toddlers did not show a high-guard position at the beginning of walking, and if they did, they repositioned to the middle-guard posture in the first few months of walking^6^. Moreover, the arm flexion increased around the second month of walking, and the dependence on arm raising decreased significantly^15^. Compared to other parameters, it is presumed that hand height changed significantly in a relatively short period at the early stage of walking. In addition, Yaguramaki et al*.*^12^ revealed that the evaluation of gait development based on walking experience was more accurate than that based on calendar age. However, the definition of gait onset differed depending on each parent. In this study, some parents had vague memories because the toddler started walking more than a year earlier. Therefore, gait development was evaluated by age based on the day of birth. For these reasons, we considered that there may be variations in the timing of gait onset and that individual changes were buried in these variations. If reliable information had been obtained regarding the day a toddler started walking, and if it would be possible to evaluate gait development by age, which is based on gait onset, hand height may have contributed to the improvement in accuracy of the multiple regression equation. In addition, the influence of noise due to external factors other than gait development may have led to these variations. In this study, trials that met the recruitment criteria were adopted even if participants had a toy in their hand or their hand was turning to their parent, who was in the same measurement room. As a result, hand height was considered an index that was more likely to change due to external factors and more difficult to stabilize compared to the other four gait parameters. In addition, since a useful index was obtained in this study using other parameters that showed more significant changes with less variations than the hand height, hand height did not contribute to improving the accuracy of the multiple regression equation. However, when, for example, the other gait parameters are limited, it is presumed that hand height may be a useful evaluation index, as it can be observed visually, and the data is collected easily compared with those for other gait parameters.

This study investigated the strength of the association between toddlers’ gait development and five gait parameters that have been reported in previous studies to be associated with gait development in toddlers. As a result, we revealed that it is possible to evaluate the level of gait development more accurately by combining several gait parameters that are strongly related to gait development, rather than assessing the gait development level with a single gait parameter. At the same time, we showed that gait development levels can be quantitatively assessed using age-based indicators. This result suggests that the age of gait development can be estimated from gait alone. If we can construct a system that quantitatively evaluates the level of individual gait development based only on gait, it may be possible to grasp the gait development status of toddlers based on objective indicators that are easy for even laymen to intuitively understand. Furthermore, this may be used as a preliminary screening and diagnostic support tool that does not require observation by experts. In this study, we focused on the gait development of toddlers. However, some toddlers may have problems with delayed gait development, such as gait disorders and abnormal gait. In addition, gait analysis of children with developmental disabilities and cerebral palsy has also been conducted^16,17^. Currently, the diagnosis of gait disorders and abnormalities is often based on observational studies by doctors and specialists and largely depends on the discerning skills of observers. However, early detection and intervention of gait disturbances and abnormalities may also reduce future risks and have more positive outcomes.

Regarding the limitations of this study, the first is the influence of the measurement environment and method. The participating toddlers were given sufficient time to adjust to the environment. However, because the measurement room is a special environment for them, their ability to capture natural gait may have been limited. In addition, they did not always walk straight on the walking path because they walked as they wanted without being taught how to walk. Therefore, from the trial of walking many round-trips on the walking path, we cut out the sections that were judged to be relatively natural and straight and evaluated the average. Analyzing gait, including meandering scenes that are common in toddlers, is a future issue for consideration. The second is bias pertaining to the participants who were recruited and whose data were analyzed. The participants of this study were limited to healthy children living near Tokyo who were collected cross-sectionally. However, whether the participant was “healthy” or not was based on the parent’s declaration and not on a physician’s diagnosis. In addition, since the residential area was restricted to the suburbs of Tokyo, the results obtained in this study are limited to data on Japanese people. Furthermore, if individual gait development can be collected longitudinally, it may be possible to clarify the individual gait development mechanisms in more detail. To examine the presence or absence of gait disorders and abnormalities, the impact on residential areas, and the acquisition of longitudinal walking data, it is necessary to collect and examine more data. Third, a method for selecting gait parameters could have been considered. In this study, we selected only five major gait parameters associated with gait development, as reported by previous studies. However, some untested gait parameters may be highly associated with gait development. In addition, although this study focused on gait development in relatively healthy toddlers, there may be other better gait parameters for evaluating the delay in gait development for toddlers with gait disorders or gait abnormalities. Therefore, selecting gait parameters in conjunction with the research objectives would be beneficial.

In conclusion, this study suggests that it may be possible to estimate the age of individual gait development with some accuracy from a toddler’s gait. This indicates the importance of assessing toddlers’ gait development, and not only grasping the developmental trend of each gait parameter but also evaluating gait as a whole while considering the strength of the relationship between each gait parameter and the toddlers’ gait development. Moreover, by combining several gait parameters highly related to gait development, the age of gait development, or the level of gait development with age as its indicator, could be estimated quantitatively from the gait of toddlers alone. The findings of this study may be used in a wide range of fields in the future, such as preliminary screening and diagnostic support tools that do not require observation by specialists and systems that monitor children’s daily gait, quantitatively evaluate developmental status, and judge health conditions.

## Methods

### Participants

Table 3 summarizes the characteristics of toddlers analyzed. Overall, 99 healthy toddlers aged 13–37 months who could walk independently participated in this study. Of these, 97 toddlers (49 boys and 48 girls) whose parents answered all survey questions, such as when they started walking, sex, expected date of birth, actual date of birth, weight at birth, gait onset period, current height, current weight, diaper usage (whether under usage or not or type or pants used), and diaper brand, were analyzed. The body height, weight, and onset age of walking of these toddlers were equivalent to those reported in previous studies of young children of similar ages^12,18^. Since previous studies^19^ have shown that it is unnecessary to consider the influence of sex differences when evaluating toddler gait and interpreting the results, this study did not perform a sex-specific analysis. The selection criteria included healthy toddlers living in the suburbs of Tokyo who can visit the examination venue with their parents. The exclusion criteria were toddlers with chronic diseases, skin symptoms such as severe dermatitis, suffering from infectious diseases, inability to be measured due to awkwardness/shyness, prone to tape rashes, and excessively reluctant to wear hats. This study was conducted in accordance with the guidelines proposed in the Helsinki Declaration, and the research protocol was approved by the Human Research Ethics Committee, Kao Corporation (research number T206-190220). Written informed consent forms were provided to the parents of the toddlers, and the test was conducted following their signing of the consent forms.

**Table 3:** Characteristics of toddlers analyzed.

N = 97 (49 boys, 48 girls)	Group	Mean	SD	95% CI		RM ANOVA		Bonferroni’s post-hoc test
				Lower	Upper	*p*	*η*^2^	
Age (months)	A	17.41	2.43	16.57	18.26	***	0.899	A < B*** A < C*** B < C***
	B	25.23	2.22	24.41	26.04			
	C	32.97	1.86	32.30	33.64			
Height (cm)	A	78.57	3.98	77.18	79.95	***	0.606	A < B*** A < C*** B < C***
	B	85.02	4.05	83.54	86.51			
	C	89.93	3.52	88.67	91.20			
Weight (kg)	A	10.46	1.29	10.01	10.91	***	0.418	A < B*** A < C*** B < C**
	B	12.04	1.47	11.50	12.58			
	C	13.24	1.39	12.74	13.74			
Onset age of walking (months)	A	5.53	2.57	4.63	6.43	***	0.866	A < B*** A < C*** B < C***
	B	12.29	2.67	11.31	13.27			
	C	21.28	2.54	20.36	22.20			

Group A, 13–21 months (N = 34); Group B, 22–29 months (N = 31); Group C, 30–37 months (N = 32). [%height] indicates the height ratio.

Data are presented as means.

SD: standard deviation, CI: confidence interval, *p*: p-value, *η*^2^: effect size and Bonferroni’s post-hoc test.

**p < 0.01; ***p < 0.001.

### Data collection

**Figure 3:**
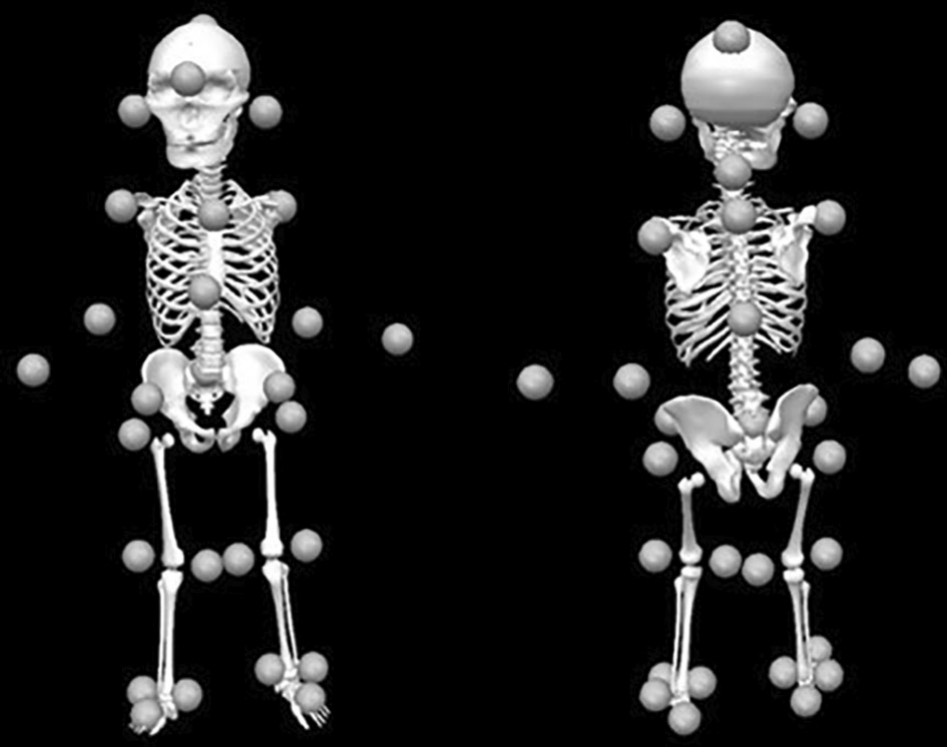
Thirty-two mounting positions of reflective markers and the body model.

The data collection method was the same as that of a previous study^20^ published by our group in 2021. The data collection method is briefly described below. Participants were asked to wear accustomed and brand-new disposable diapers (without being soiled) and swimming caps. In that state, a measuring reflective marker was affixed to the body. Figure 3 shows the mounting position of the reflective marker for measurement. The measurement reflective markers were attached to the head (between the eyebrows, top of the head, occipital, and tragion), upper limbs (the acromion, elbow, wrist, upper margin of sternum, vertebra prominens, xiphoid process, and the 10th thoracic vertebra), pelvis (superior anterior iliac spur, sacrum), and lower limb (great trochanter, inner knee, outer knee, medial malleolus, lateral malleolus, heel, and toe). A three-dimensional (3D) motion analyzer (Vicon Vantage V5, 100 Hz; Vicon, Oxford, UK) consisting of 12 infrared reflective cameras was used for the measurement. Each participant walked barefooted independently at his/her own time on a walking path of approximately 10 m more than three times (round trip) without being taught about walking speed or step length. On of the approximately 10-m walking path, since reflective markers tend to enter the camera’s blind spot at about 2 m at both ends, the section that could actually be analyzed was about 6 m in the center of the walking path. Attempts in which the toddler stopped walking, turned around, ran, or fell were excluded. However, in this study, unlike our aforementioned study in 2021^20^, multiple measurement conditions were not set and saline solution was not injected into disposable diapers to simulate urination.

### Data analysis

The data analysis method was the same as that of the previous study^20^ published by our group in 2021. Noise was removed by applying a fourth-order Butterworth filter to the raw data of the measured marker 3D coordinates. The walking cycle, step length, walk ratio, and each marker coordinate were calculated as gait parameters. Visual 3D software (C-Motion Inc., Rockville, MD) was used for filtering and variable calculations. The origin of the marker coordinates was defined as the center of the participant’s pelvis (PV) in the laboratory coordinate system. The spatial coordinates of the recorded markers were defined as the *x*-axis in the front-rear direction, *y*-axis in the left–right direction, and *z*-axis in the vertical direction with respect to the participant’s traveling direction. Furthermore, each marker coordinate was time-normalized by cutting out the left and right one-step cycle from one heel grounding to the next. In this study, data of four or more walking cycles in total of both left and right sides were extracted and averaged for each participating toddler from sections that they walked relatively naturally straight by visual check and did not have any defects of reflective markers.

This study applied five gait parameters: walk ratio, hand height, trunk sway, knee-to-knee distance, and waist height. As criteria for selecting gait parameters, we considered (1) those previously reported in multiple studies, (2) easy to evaluate with observation by amateurs, and (3) small influence of definition. The definition and calculation method of each gait parameter is shown below.

- Walk ratio [cm/steps/min]: step length [cm]/cadence [steps/min].
- Hand height: *z*-axis maximum value from PV to each wrist.
- Trunk sway: *y*-axis amplitude value of the base of the neck.
- Knee-to-knee distance: *y*-axis maximum value on the outside of the right knee – *y*-axis minimum value on the outside of the left knee.
- Waist height: *z*-axis median value from PV to each heel.

Since eligible toddlers of this study were experiencing not only developing gait but also significant physical changes, to minimize the effect of height, four gait parameters excluding the walk ratio were divided by height and multiplied by 100 to calculate the height ratio (%height)^21,22^.

### Statistical analysis

The association between each gait parameter and age was evaluated using Pearson’s correlation coefficient. Measurements of characteristics and gait parameters for each age group were assessed using one-way ANOVA and Bonferroni post hoc analysis. An estimation model of walking age using five gait parameters was created by multiple regression analysis. The walking data of 97 participants were randomly divided into training data (80%, N = 78) and test data (20%, N = 19)^23,24^. Table 4 shows the characteristics of the training and test datasets. Age, height, weight, and months of walking experience were not significantly different between the training and test datasets. Using the training data, we created an estimation model using three methods: forced entry, backward elimination, and forward inclusion, with age as the dependent variable and five gait parameters as independent variables. The accuracy of the estimation model was verified using test data. Statistical significance was set at *p* = 0.05, 0.01, and 0.001 for both the correlation and multiple regression analyses. All statistical analyses were performed using SPSS ver. 28.0 (IBM, Armonk, NY).

**Table 4:** Characteristics of the training and test datasets.

Variable	Training (N = 78)		Test (N = 19)		*p*
	Mean	SD	Mean	SD	
Age (months)	25.1	6.5	24.5	8.2	0.743
Height (cm)	84.4	5.6	84.3	7.8	0.943
Weight (kg)	11.9	1.7	11.9	2.3	0.919
Onset age of walking (months)	12.9	6.7	12.8	8.3	0.976

Data are presented as means.

SD: standard deviation, *p*: p-value.

## Data Availability

The datasets presented in this article are not readily available due to participants’ confidentiality concerns. To access the datasets, a reasonable request should be directed to the corresponding author.
